# Individual Differences in Frustrative Nonreward Behavior for Sucrose in Rats Predict Motivation for Fentanyl under Progressive Ratio

**DOI:** 10.1523/ENEURO.0136-21.2021

**Published:** 2021-10-22

**Authors:** Tileena E. S. Vasquez, Poonam Shah, Jessica Di Re, Fernanda Laezza, Thomas A. Green

**Affiliations:** 1 Department of Pharmacology and Toxicology; 2 Neuroscience Graduate Program; 3 Center for Addiction Research; 4 Mental Health Research Group, The University of Texas Medical Branch, Galveston, TX, 77555-0519

**Keywords:** aggression related, craving, drug abuse, motivation, opioid, progressive ratio

## Abstract

Frustrative nonreward (FN) is a construct in the Negative Valence Systems domain of the Research Domain Criteria (RDoC) from the National Institute of Mental Health. An organism’s response to frustrating situations (e.g., inability to obtain an expected reward) has broad implications for a variety of neuropsychiatric conditions, including substance use disorders. The current project developed a first of its kind rat operant behavioral model of FN based loosely on the human Point Subtraction Aggression Paradigm (PSAP). The current study shows that individual differences in FN for sucrose pellets are consistent across sessions at baseline and that the task is sensitive to reward size in male rats. More importantly, high FN behavior for sucrose predicts early “breaking” for intravenous fentanyl self-administration under a progressive ratio (PR) schedule. These results solidify frustration/ FN as an important factor for substance use disorders in addition to craving, impulsivity, and habit.

## Significance Statement

There are a few human studies that suggest that frustration behavior is a risk factor for substance use disorders. The present study focuses on rats’ behavior during frustrative nonreward (FN) tasks in substance abuse-related operant procedures. These procedures used bar press durations which have been shown to be a measure of frustration-like behavior. Accordingly, the study found that individual differences in frustration-like behavior can be used predict drug seeking and taking. Thus, the results of this study support frustration as a fourth major facet of substance abuse-related behavior, adding to craving, impulsivity, and habit.

## Introduction

Historically, animal models of substance use disorders have focused on facets such as craving, impulsivity, or habit. We propose an animal model to study another facet of substance use disorder-related behavior: that of frustration. Previous research showed that rats increase lever-press durations under conditions of frustration for drug or sucrose reward (Vasquez et al., 2021). Our data show that this measure of frustration is a robust, replicable, and sensitive surrogate for frustration behavior.

While there is little research into the role of frustration in substance use disorders specifically, a few studies highlighted that persons with substance use disorders rate higher in tests of frustration and that sensitivity to frustration correlates with number of relapses (Baars et al., 2013; Ramirez-Castillo et al., 2019). The limited number of rodent models generally support the human research, demonstrating frustration from loss of an alternative food reinforcer increases drug seeking and taking (Podlesnik et al., 2006; Quick et al., 2011; Gipson et al., 2012; Pyszczynski and Shahan, 2013; Ginsburg and Lamb, 2018), likely an expression of negative urgency (Gipson et al., 2012).

Moreover, previous studies that exclusively focused on frustration used slower speeds running down a runway as a measure of frustration behavior by using food solely as the reinforcement (Amsel and Roussel, 1952; Adelman and Maatsch, 1955; Capaldi, 1974). Additional studies investigated arousal of frustration and associated cues in nonreinforced or noncontinuous reinforcement conditions, referring to these conditions as instances of nonreward (Amsel, 1958; Daly, 1974). The current project expands on these techniques with a lever-press operant response procedure to study the effects of frustration in drug self-administration.

In addition to substance use disorders, frustration is a significant component of many other neuropsychiatric conditions ranging from conduct disorder to personality disorders to mood disorders (Jeronimus et al., 2016, 2017; Jeronimus and Laceulle, 2018). The National Institute of Mental Health currently classifies frustrative nonreward (FN) as a construct in the Negative Valence Systems domain of the Research Domain Criteria (RDoC) framework. An organism’s appropriate response to a frustrating situation (i.e., being unable or having to work harder to fulfill a goal) is an important aspect of normal behavior, and inappropriate responses to frustration can be a component of a neuropsychiatric condition.

The objective of this project was to develop a FN operant task, based loosely on the human point subtraction aggression paradigm (PSAP), that can be used as a tool to identify rat’s individual differences in frustration-like behavior during self-administration of sucrose pellets with the hypothesis that those individual differences can predict a rat’s drug seeking or taking before exposure to the drug. The human PSAP is a validated behavioral measure of aggression in response to perceived provocation and (Cherek et al., 1997a,b) subjects had the option to respond in one of three ways to obtain points: the first option was to continue normal responding to earn points (nonaggressive responding), the second option was to subtract points from a fictious person to add to their own score (retaliation/aggressive responding), and the last option was to protect points from being subtracted (escape). Rats are incapable of comprehending instructions of a conspecific in the next cage stealing points, but it has been shown that rats are capable of knowing how close they are to receiving a reinforcer through conditioning of reward expectancy (Amsel, 1958; Daly, 1974).

The current study is the first lever-press operant-based paradigm for quantifying FN in rats. Here, we show that the task is consistent at baseline across days, responsive to reward size, and that low, medium, and high frustration behavior for sucrose reward predict early breaking on a progressive ratio (PR) schedule for intravenous fentanyl. The break point during PR is when the response output falls below a predefined level and is commonly used to evaluate the reinforcing efficacy of abused drugs (Cain and Bardo, 2010). Break point is generally defined as the last ratio in effect when the rat fails to meet the response output requirements for that ratio. However, our data use an alternative measure of break point: the total number of reinforcing events during the session, which is slightly different from the last ratio (Roberts, 2010).

Additionally, prior research shows that manipulations of retinoic acid signaling alter drug taking and seeking (Zhang et al., 2016; Crofton et al., 2021); thus, we hypothesized that overexpression of retinoic acid receptor (RAR)β in the nucleus accumbens shell (shNAc) would alter FN and/or fentanyl taking/seeking, but this hypothesis was not supported by the data.

## Materials and Methods

### Animals

Male Sprague Dawley rats were obtained from Envigo at 225–250 g. Except during food regulation, rats were pair-housed throughout the experiments and maintained in a controlled environment (temperature, 22°C; relative humidity, 50%; and 12/12 h light/dark cycle, lights on 6 A.M.) in an Association for Assessment and Accreditation of Laboratory Animal Care (AAALAC). Procedures were approved by the The University of Texas Medical Branch Institutional Animal Care and Use Committee and conform to the National Institutes of Health *Guide for the Care and Use of Laboratory Animals*.

### Sucrose operant responding: initial training

Rats (*n* = 20) were initially placed on a regulated intake diet for 6 d until rats reached 85% of free-feed body weight. Rats were then placed in operant chambers where achieving the response requirement on the active lever resulted in the extinguishing of the house light, the illumination of two circular cues lights located above the levers for 5 s, delivery of a banana-flavored sucrose pellet (45 mg; Bio-Serv). The illumination of the two cue lights signaling the delivery of the renforcer also serve to signal a time-out during which responding during the 5 s was recorded but animals could not earn more sucrose. Throughout the session, responding on the inactive lever was recorded but had no consequences.

#### FR responding

During the first session rats were allowed to perform a single lever press (FR1) to receive sucrose pellets for 2 h with an unconditional pellet provided every 10 min until they self-administered 100 pellets. Rats that showed slower learning (e.g., did not reach 100 pellets) were placed in the operant chambers again until they could self-administer a combined 100 pellets across the sessions. Next, rats were placed on an FR1 schedule for 15 min, and after four sessions on FR1, the response requirement for the next session was increased to FR3 (three lever presses to receive the reinforcer), followed by an increase to FR5 the subsequent four sessions.

#### Cued extinction

The protocol consisted of 2 h with cue lights delivered under the normal FR5 schedule but no sucrose pellet delivery. The extinction session was immediately followed by 15 min of maintenance sucrose self-administration at an FR1 schedule to prevent extinction from affecting the next session.

#### PR

For the next three sessions the rats were placed on a PR schedule in which each successive reinforcement required an increasing number of lever-press responses according to the following semi-logarithmic progression, 1, 2, 4, 6, 9, 12, 15, 20, etc. The session continued until the rats went 1 h without obtaining a reinforcement, or up to a maximum of 6 h.

### The FN sucrose task

The FN task is an operant lever-press procedure based loosely on the PSAP in humans (Cherek et al., 1997a,b). The training procedure consists of a compound schedule where the two cue lights and the house light are illuminated at the beginning of the trial, and rats press the active lever two times to turn off the left cue light (leaving two lights on), two more times to turn off the right cue light, two more to turn off the house light, and two more for delivery of the reward (FN8 = eight presses for a reward), when all lights are again illuminated and the rat can begin the next trial. There was no time-out, and the next trial began immediately, therefore there was no illumination of the two circular cue lights concurrent with the delivery of the reinforcement. Each bar press during a trial was recorded as a point to be added to the rat’s score for the trial. Once the rat achieved the required score (e.g., FN8 required a score of eight points per trial) the points were reset for the next trial. Thus, the more points the rat has, the less light in the chamber. For training, each session the number of presses was incremented until five points per step (FN12, FN16, and then FN20). Thus, the FN20 procedure requires 20 lever presses per reward (FN20 no frustration; five presses to turn off each light). Light cue contingencies for the FN task were different from the FR tasks (sucrose and drug) to help the rats discriminate between the two tasks. For data analysis, five rats were removed from the study because of non-acquisition of the FN task.

#### Determining sensitivity to reward magnitude

Rats were first given “FN20 no frustration” throughout the first session with only one pellet per trial. For the next session, the first five trials were one pellet and the subsequent trials were four pellets (incentive upshift). The next session was four pellets per trial throughout, and the last session was four pellets for the first five trials and one pellet for all remaining trials (incentive downshift). Only data from after the sixth trial were analyzed.

#### Adding the frustration component

To introduce a frustration element, when the rat presses for the 18th point of the FN20, instead of incrementing the score by one point, the computer can deduct seven points as programmed, bringing the point level from 17 to 10, turning on the house light to signal deducted points. The rat must continue to press to make up the lost points. For the “FN20 low frustration” condition, seven points are deducted every other trial (i.e., 27 presses for the deduction trial). For the “FN20 medium frustration” condition, seven points are deducted twice every other trial (i.e., 34 responses for deduction trial). For the “FN20 high frustration” condition, seven points are deducted three times each trial (41 responses for every trial), and the “FN20 extreme frustration” deducts seven points 26 times for each trial, requiring 202 presses for each reward. One session of “FN20 no frustration” intervened between each of the FN20 low, medium, and high condition to maintain stable responding. A “frustration score” was calculated as the average lever press duration of each frustration session (FN low, medium, high, and extreme) divided by the average lever press duration of that subject’s “no frustration” condition (FN20 none).

### Fentanyl operant responding

After one week of free feed in the colony room, rats were injected bilaterally into the shNAc with 1 μl of adeno-associated viral vector expressing GFP or one expressing GFP plus the RARβ. Coordinates were AP = 1.3, L = 2.4 from bregma and DV = −6.7 mm from dura (Zhang et al., 2014). The shNAc was targeted as the repetitive activation of the shNAc by drugs of abuse results in strengthening of stimulus-reward and stimulus-response associations (Di Chiara, 1998, 2002; Di Chiara et al., 2004). Additionally, retinoic acid signaling is the most enhanced shNAc pathway with RARβ being one transcript that was identified as a strong target (Zhang et al., 2016; Crofton et al., 2021). It is also suggested that the shNAc plays a role in goal-oriented behavior (Mannella et al., 2013), and it is generally understood that the experience of frustration occurs when the goal is denied or made more difficult to achieve. Therefore, the original hypothesis was that decreasing RARβ in the shNAc would alter drug seeking and frustration behavior. However, this hypothesis was not supported by the data as there was no significant effect on fentanyl self-administration behavior. Thus, when analyzing these data for number of infusions and bar press durations the animals were collapsed into one group.

After one week of recovery, rats were anesthetized with ketamine (100 mg/kg, i.p.) and xylazine (10 mg/kg, i.p.) and implanted with indwelling intrajugular SILASTIC catheters as described previously (Zhang et al., 2016; Crofton et al., 2017). To maintain catheter patency, catheters were flushed daily with 0.1 ml of heparinized (10 U/ml) saline with ticarcillin (0.067 *g*/ml). Following one-week recovery from catheter surgery, animals were placed in the operant chambers to self-administer fentanyl HCl (0.0032 mg/kg/infusion; NIDA Drug Supply Program).

#### FR responding

Animals began fentanyl self-administration on a continuous schedule (FR1) of reinforcement until they were responding consistently for 4 d (>10 infusions per session and less than a difference of 10 infusion variability in daily intake). Each session lasted 3 h where a single response on the active lever resulted in a 0.1-ml intravenous infusion delivered over 5.8 s, concurrent with the illumination of two circular cues lights located above the levers. Each infusion was followed by a 20-s time-out period during which the cue lights remained illuminated, the house light was extinguished, and responding was recorded but animals could not earn more fentanyl. The cued time-out period was extended to 20 s from the 5 s during sucrose operant responding to prevent rats from potentially overdosing. Throughout the session, responding on the inactive lever was recorded but had no consequences.

#### Cued extinction

After stabilization on FR1, the response requirement for the next three sessions was a between-session cued extinction procedure consisting of 3 h with cue lights was delivered under the normal FR1 schedule but with no drug delivery. To prevent full withdrawal, the extinction session was immediately followed by 1 h of maintenance fentanyl self-administration at an FR1 schedule.

#### PR

The next three sessions the rats were placed on a PR schedule in which each successive fentanyl injection (0.0032 mg/kg/infusion) required an increasing number of lever-press responses according to the following semi-logarithmic progression, 1, 2, 4, 6, 9, 12, 15, 20, etc. (Green et al., 2002). The session continued until the rats went 1 h without obtaining a reinforcer, or up to a maximum of 6 h.

### Statistical analysis of behavior

For estimation based on confidence intervals (CIs), we directly introduced the raw data in https://www.estimationstats.com/ and downloaded the results and graphs for the permutation *t* tests in which 5000 bootstrap samples were taken; the CI is bias-corrected and accelerated (Ho et al., 2019; Manouze et al., 2019). The *p* values reported are the likelihoods of observing the effect sizes, if the null hypothesis of zero difference is true. For each permutation *p* value, 5000 reshuffles of the control and test labels were performed. The effect sizes and CIs are as: effect size [CI width lower bound; upper bound]. Simple linear regression was used to assess correlations. The α level was set at *p* < 0.05. There was no correction for multiple comparisons. Rats not completing a given experiment were not considered in that analysis. FN data analysis, five rats were removed from because of non-acquisition, making the *N* for this analysis 15. Additionally, one rat was removed from fentanyl self-administration analysis for unstable responding during acquisition, making the final *N* for the study 14.

## Results

### Consistency of FN20 responding

**Figure 1.**
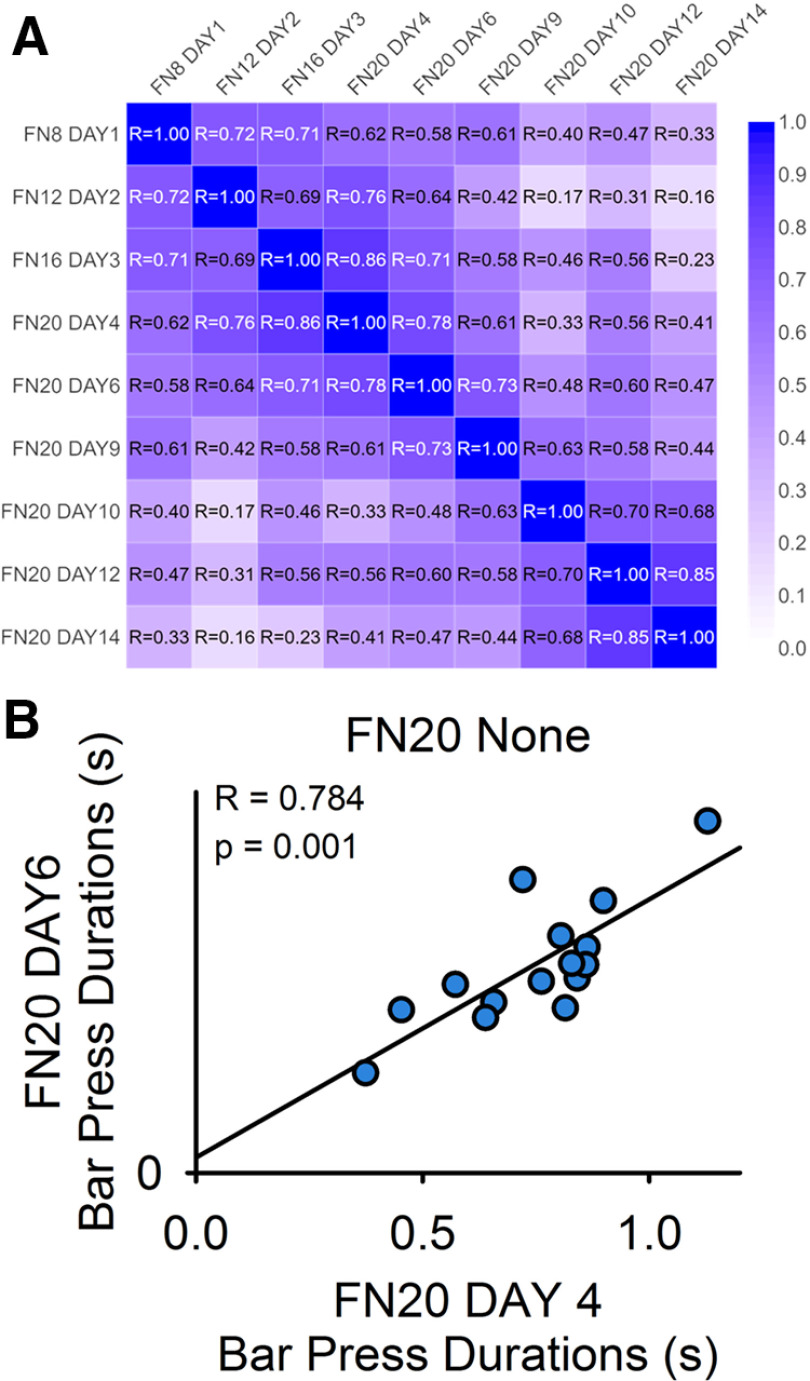
Frustration level is consistent across FN sessions. ***A***, Correlation matrix with heat map of the correlation coefficients (*R*) from simple linear regression analyses to investigate average lever press durations compared across multiple sessions of FN training and stabilization (i.e., no frustration trials). Blue represents strong positive correlation and white represents no correlation. ***B***, Representative simple linear regression analysis of average lever press durations during FN20 day 4 versus FN20 day 6.

There were strong positive correlations when comparing among session average lever press durations during training and stabilization sessions for FN responding with no frustration trials (Fig. 1*A*). A representative scatterplot of FN20 day 4 versus FN20 day 6 is shown in Figure 1*B* (*R* = 0.784, *p* = 0.001). This demonstrates that responding is surprisingly stable across FN no frustration sessions. The exceptions were from comparisons from early training or those that had the greatest number of intervening days (e.g., FN12 day 2 vs FN20 day 14, *R* = 0.16, *p* = 0.590). Active:inactive lever press ratio was >10:1 for all rats.

### Sensitivity to reward magnitude

**Figure 2.**
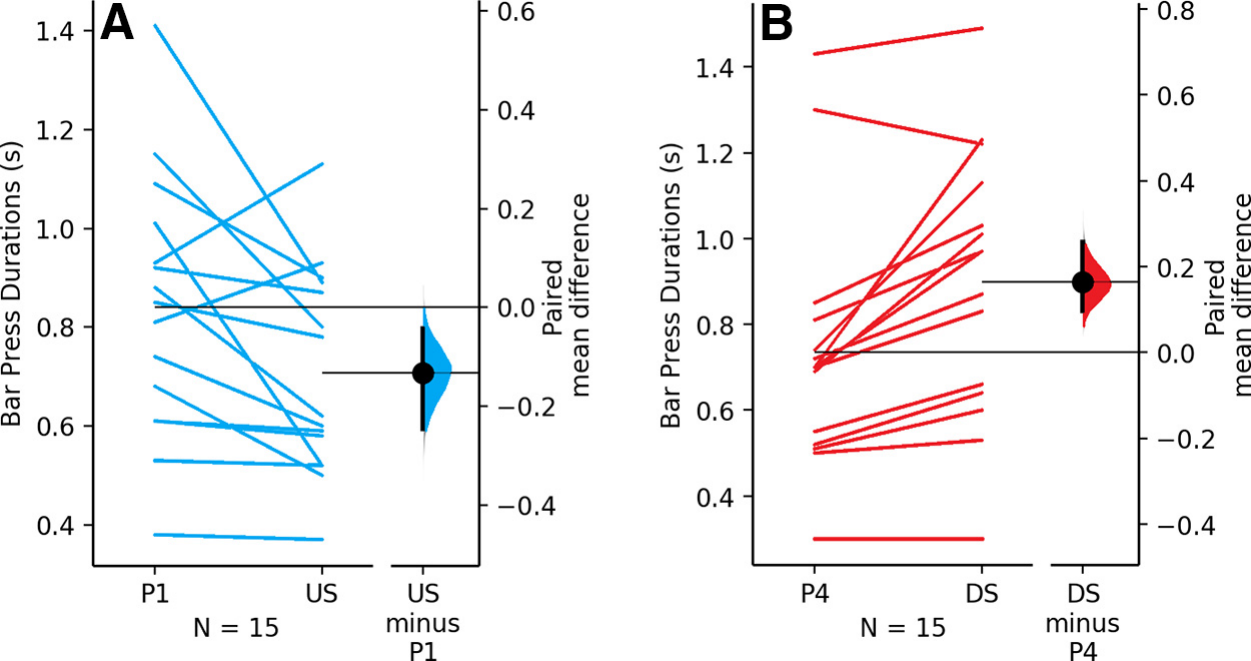
Frustration level is sensitive to reinforcer size. ***A***, The paired mean difference between average lever press durations (seconds) during the sucrose self-administration session for one pellet throughout the session (P1) and incentive upshift (one pellet for the first five reinforcers and four pellets for all subsequent reinforcers; US) is shown in the above Gardner–Altman estimation plot. Both groups are plotted on the left axes as a slope graph: each paired set of observations is connected by a line. The paired mean difference is plotted on a floating axes on the right as a bootstrap sampling distribution. The mean difference is depicted as a dot; the 95% CI is indicated by the ends of the vertical error bar. ***B***, The paired mean difference between average lever press durations (seconds) for four pellet throughout the session (P4) and incentive downshift (four pellet for the first five reinforcers and one pellets for all subsequent reinforcers; DS) is shown in the above Gardner–Altman estimation plot.

Rats demonstrated a significant decrease in average lever press durations when the reward size was changed mid-session from one to four pellets (incentive upshift) compared with the previous session of one pellet throughout. The paired mean difference between one pellet and upshift is −0.133 [95.0% CI −0.245, −0.044]. The *p* value of the two-sided permutation *t* test is 0.0214 (Fig. 2*A*). There was also a significant increase in average lever press durations when the reward size was changed mid-session from four pellets to one pellet (incentive downshift) compared with the previous session of four pellets throughout. The paired mean difference between four pellet and downshift is 0.164 [95.0% CI 0.098, 0.257]. The *p* value of the two-sided permutation *t* test is 0.0002 (Fig. 2*B*).

### Individual differences in sucrose FN frustration responding versus PR fentanyl infusions

**Figure 3.**
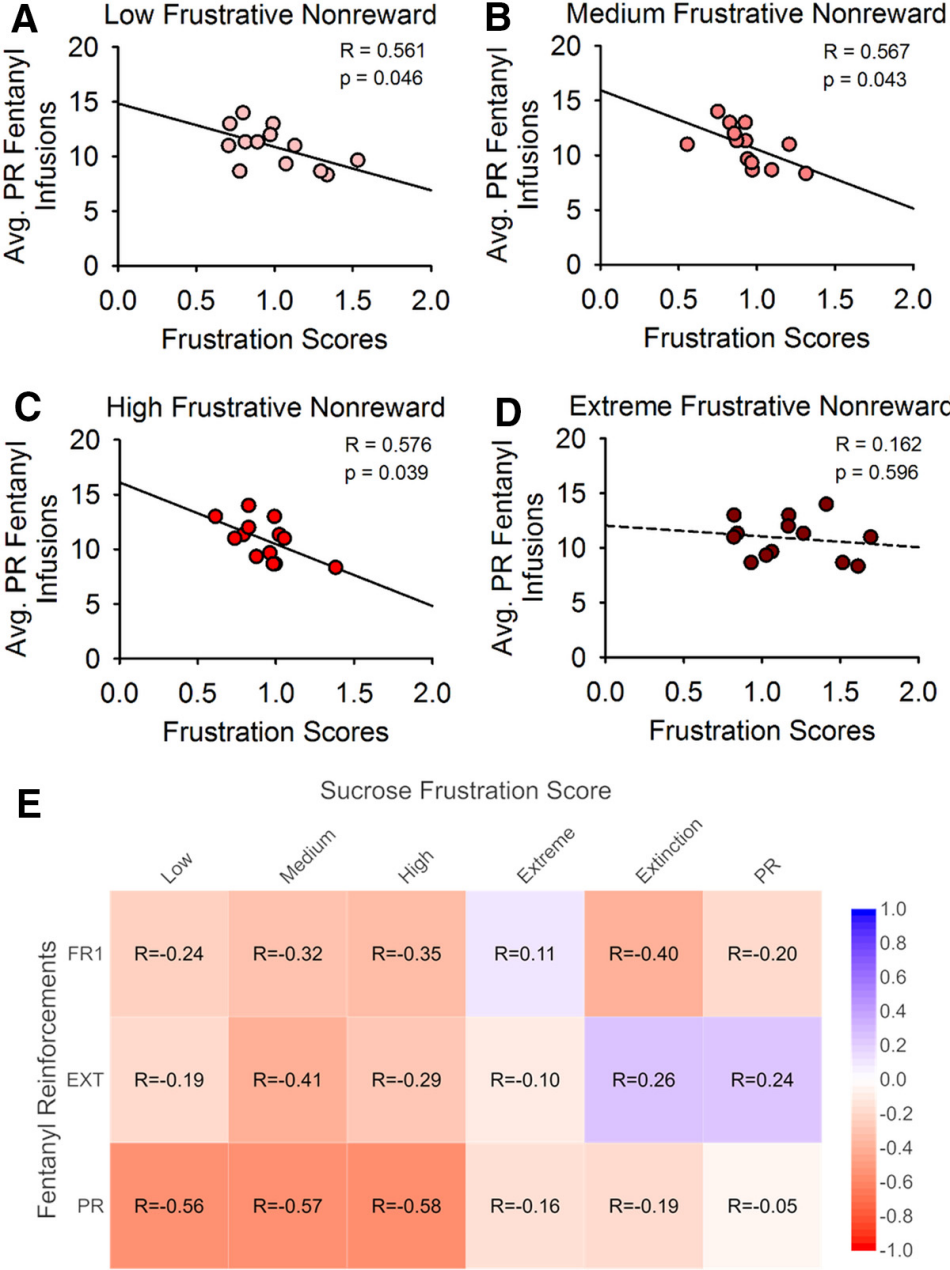
Frustration scores predict break point for PR. ***A***, Simple linear regression was used to investigate the relationship between frustration score during low FN sucrose self-administration and the average number of infusions during fentanyl PR. ***B***, Relationship between frustration score during medium FN sucrose self-administration and the average number of infusions during fentanyl PR. ***C***, Relationship between frustration score during high FN sucrose self-administration and the average number of infusions during fentanyl PR. ***D***, Relationship between frustration score during extreme FN sucrose self-administration and the average number of infusions during fentanyl PR. ***E***, Correlation matrix with a heat map of the correlation coefficients (*R*) from simple linear regression analyses to investigate relationship of frustration scores during the sucrose FN tasks, extinction, and PR with the average number of fentanyl infusions during FR1, EXT, and PR. Blue represents strong positive correlation, white represents no correlation, red represents strong negative correlation.

The goal of this project was to determine whether seeking or taking of fentanyl could be predicted by an individual rat’s frustration-like behavior for sucrose pellets before exposure to the drug. Thus, frustration scores for conditions of FN low, medium, high, and extreme frustration were used to quantify each rat’s frustration score, and these were compared with their number of reinforcements during fentanyl self-administration sessions. Frustration scores for sucrose low, medium, high, extreme FN, extinction, and PR were compared with the number of fentanyl reinforcements earned during FR1, extinction, and PR. Of these comparisons there were only significant strong correlations for low, medium, and high frustration scores for PR fentanyl infusions (averaged across three sessions; Fig. 3*E*). The statistically significant negative correlations of low, medium, and high frustration scores with average PR fentanyl infusions were *R* = 0.561, *p* = 0.046 for low (Fig. 3*A*), *R* = 0.567, *p* = 0.043 for medium (Fig. 3*B*), and *R* = 0.576, *p* = 0.039 for high (Fig. 3*C*). Extreme Frustration scores, however, did not significantly correlate with average PR fentanyl infusions (*R* = 0.162, *p* = 0.596; Fig. 3*D*). Additionally, extinction, and PR scores also did not significantly correlate with average PR fentanyl infusions (extinction score, *R* = 0.187, *p* = 0.459; PR score, *R* = 0.054, *p* = 0.832; data not shown).

## Discussion

High frustration has been shown to predict an increased risk to develop anxiety, depression, substance abuse, and thought disorders (Jeronimus et al., 2016, 2017; Jeronimus and Laceulle, 2018). This animal study expands on the role of frustration in substance use disorders by creating a FN task constructed using the same concept as the human PSAP. The FN task demonstrates that individual differences in a rat’s frustration level are consistent throughout baseline FN conditions and are sensitive to reward magnitude. Most interestingly, this study of FN demonstrates that individual differences in FN sucrose pellet self-administration can be used to predict a rat’s motivation for intravenous fentanyl self-administration under a PR schedule. Accordingly, rats with higher frustration scores during low, medium, and high sucrose FN conditions (but not extreme FN, extinction, or PR for sucrose) obtain fewer infusions of fentanyl during PR. Interestingly, these data also demonstrate that to be able to predict a rat’s intake of fentanyl during PR using frustration scores, the frustration difficulty during the FN task must not be extreme. This is likely a ceiling effect where nearly all animals show high frustration scores, thus washing out individual differences in frustration scores.

In a longitudinal human study by Jeronimus et al. (2017), data showed that high frustration in adolecence predicted increases in externalizing symptoms of psychopathology like drug use, suggesting that frustration behavior is a risk factor for substance use disorders. Our data would predict the opposite. However, it is important to understand that “frustration” is not a unitary phenomenon. The type of frustration of Jeronimus’ study was parental report of a child’s irritability and aggression. The type of frustration measured by lever press durations is related to extinguishing responding for a reinforcer. Recent research demonstrated that lever press durations can be used as a measure of frustration level (Vasquez et al., 2021), and rats exhibited their longest lever press durations late in extinction sessions or shortly before breaking under a PR schedule for drug. Correlations of frustration scores for sucrose FN tasks versus PR fentanyl infusions in Figure 3 demonstrate that breaking under a PR procedure for fentanyl can be predicted by individual differences in FN *sucrose* responding.

The schedules used for incentive upshift and downshift in FN responding were inspired by changes in running speed down a runway in Capaldi’s runway paradigm (Capaldi, 1974). Our data showed that durations were sensitive to reward magnitude as incentive upshift decreased durations and downshift increased durations.

This study shows that individual differences in frustration behavior for sucrose predict subsequent early breaking on a PR schedule for intravenous fentanyl. This builds on a significant foundation on individual differences research typified most clearly by Piazza and colleagues showing that high locomotor responders during exposure to a novel environment take amphetamine more readily than low responders (Piazza et al., 1989). Of relevance to the current project, two studies showed that high sucrose intake during free access predicted amphetamine and cocaine taking (DeSousa et al., 2000; Gosnell, 2000). The current study found no such link with operant sucrose intake failing to predict fentanyl intake, but rather frustration to sucrose responding predicting fentanyl early breaking.

It should be noted that the low, medium, and high FN scores predicting PR breaking is correlational and should be further investigated in a causal fashion. Future studies will affect neurobiological aspects of frustration to determine the underlying mechanisms of the effect of FN on motivation in substance abuse related behavior in rats.

Our conclusion is that a rat’s frustration level is a consistent trait and that increased sensitivity to frustration can be used to predict a rat’s motivation to seek fentanyl. Thus, these FN tasks provide a novel tool to assess individual differences in a rat’s frustration levels that can be used in future studies of frustration/FN as an important factor for substance use disorders in addition to craving, impulsivity, and habit.

## Synthesis

Reviewing Editor: Alexxai Kravitz, Washington University in St. Louis Decisions are customarily a result of the Reviewing Editor and the peer reviewers coming together and discussing their recommendations until a consensus is reached. When revisions are invited, a fact-based synthesis statement explaining their decision and outlining what is needed to prepare a revision will be listed below. The following reviewer(s) agreed to reveal their identity: Jonathan Britt.

The reviewers and I all commented on how responsive this revision was to the prior reviews, and how it greatly improved in all aspects. One issue still generated discussion, which was the issue about the RARβ viral infusions. In the last round they were mentioned in the methods but not in the rest of the paper. Now it is more clear what happened with these infusions but the results fall into a nebulous area, as they produced a trend towards an effect on one measure, but it is not entirely clear why this effect is weak (or potentially non-existent). Reviewer 1 brought up several critiques noting that without showing histology or ideally a functional verification that the viral manipulation worked as expected, it’s not fair to conclude there was “No Effects of Over Expression of RARβ in the NAc Shell", as the Title of Table 1 suggests. This reviewer also brought up some methodological concerns with this experiment. I am appending the full comments from Reviewer 1 below, but what we decided was a good solution was to either 1) address these comments if you have data that the viral manipulation worked as expected and can demonstrate it; or 2) remove these data from the results, but explain the rationale for and presence of the virus and the lack of behavioral effect in the methods, hence combining the groups for analysis. Hopefully this can be done in a non-awkward way that enhances the completeness of the manuscript without introducing potentially incomplete data. To be clear, both reviewers thought the manuscript was greatly improved and should now be published. As such, I do not plan to send it back out for review, but I think this point is important to resolve.

Reviewer 1 had one other minor point about the intro, pasted below.

**************************

Reviewer 1

**************************

1. In the introduction, line 40 (page 2) states “However, there are many studies that focus exclusively on frustration using food as the reinforcement” before citing runway studies. Yet, the current paper uses food as a reinforcer. Thus, this should be restated, as it sets up the reader to think that using food as a reinforcer is problematic, and this is somehow a gap in the rigor of prior research that this current study is going to address. Given that food is used in this current study as well, this sentence should be revised.

2. There is no histological verification of NAshell viral hits or successful manipulation of receptor expression levels.

Given the lack of effects with the overexpression experiment, this seems like a rather large omission. If overexpressing a protein, western blot or PCR should be performed to assess expression patterns in control vs. active constructs. Further, there are no methods for (1) how constructs were developed (is this a shRNA packaged in a AAV? what serotype of AAV? Where were the constructs developed? etc), (2) whether they were BSL-1 or BSL-2, (3) how viral constructs were validated?

It is important to publish negative data, however, without rigorously verifying that these viruses alter expression of the protein of interest, and without verifying that the region of interest was accurately targeted in each animal, these data are problematic to include. An alternative explanation, for example, could be that the shell wasn’t consistently targeted, or the constructs didn’t work to alter protein expression, and that could underlie the negative effects reported here.

3. The discussion makes no mention of the negative NAshell data. There should be discussion regarding potential reasons why this experiment failed to produce significant results. For example, is it that the shell isn’t involved in FN? Are there other brain regions that may be involved? Is this specific to the shell subcompartment, or could the core be involved? Is the amygdala involved? Why was there no effect with this particular protein? Why would overexpression be the way to manipulate expression (in other words, why not knock it down?)? None of these points are clear in the manuscript. It would be helpful in the introduction if the authors made clear what effects were found in the citations provided, and if a clear hypothesis was developed and tested here.

4. Further, the authors target a specific protein, but there is very little in the introduction regarding why this specific protein was hypothesized to play a role here. It is also not clear why the shell was targeted. The lack of effects in the shell call into question if there could be other regions involved.

they explain what happened more fully, but their incompleteness introduces additional caveats that Dr. Gipson brings up. Also, looking closer they mention that the virus did produce a trend towards lower bar press duration in the extinction condition (p=0.05), which is their primary endpoint for frustrative behavior. So I don’t know the best way forward here. Given Dr. Gipson’s other critiques of this experiment and the lack of confirmation that the virus worked as expected, and also Dr. Britt’s potential explanation that they may themselves have concerns about this experiment but don’t want to throw out the baby with the bathwater, maybe we suggest that (assuming we’re correct that they also have doubts), they again remove it from the results and simply leave the line in the methods to explain that mice received viral infusions but they did not produce significant effects on behavior and function was not verified, so they combined them?

I had one other minor comment I was thinking to include - do you think this comment in the significance is overstating?
